# Improvement of prediction ability by integrating multi-omic datasets in barley

**DOI:** 10.1186/s12864-022-08337-7

**Published:** 2022-03-12

**Authors:** Po-Ya Wu, Benjamin Stich, Marius Weisweiler, Asis Shrestha, Alexander Erban, Philipp Westhoff, Delphine Van Inghelandt

**Affiliations:** 1 Institute of Quantitative Genetics and Genomics of Plants, Heinrich Heine University, Düsseldorf, 40225 Germany; 2 Cluster of Excellence on Plant Sciences (CEPLAS), Heinrich Heine University, Düsseldorf, 40225 Germany; 3 Department of Molecular Physiology, Max-Planck-Institute of Molecular Plant Physiology, Potsdam-Golm, 14476 Germany; 4 Institute of Plant Biochemistry, Heinrich Heine University, Düsseldorf, 40225 Germany

**Keywords:** Barley, Deleterious SV, Transcriptome, Metabolome, Genomic prediction, Omic prediction

## Abstract

**Background:**

Genomic prediction (GP) based on single nucleotide polymorphisms (SNP) has become a broadly used tool to increase the gain of selection in plant breeding. However, using predictors that are biologically closer to the phenotypes such as transcriptome and metabolome may increase the prediction ability in GP. The objectives of this study were to (i) assess the prediction ability for three yield-related phenotypic traits using different omic datasets as single predictors compared to a SNP array, where these omic datasets included different types of sequence variants (full-SV, deleterious-dSV, and tolerant-tSV), different types of transcriptome (expression presence/absence variation-ePAV, gene expression-GE, and transcript expression-TE) sampled from two tissues, leaf and seedling, and metabolites (M); (ii) investigate the improvement in prediction ability when combining multiple omic datasets information to predict phenotypic variation in barley breeding programs; (iii) explore the predictive performance when using SV, GE, and ePAV from simulated 3’end mRNA sequencing of different lengths as predictors.

**Results:**

The prediction ability from genomic best linear unbiased prediction (GBLUP) for the three traits using dSV information was higher than when using tSV, all SV information, or the SNP array. Any predictors from the transcriptome (GE, TE, as well as ePAV) and metabolome provided higher prediction abilities compared to the SNP array and SV on average across the three traits. In addition, some (di)-similarity existed between different omic datasets, and therefore provided complementary biological perspectives to phenotypic variation. Optimal combining the information of dSV, TE, ePAV, as well as metabolites into GP models could improve the prediction ability over that of the single predictors alone.

**Conclusions:**

The use of integrated omic datasets in GP model is highly recommended. Furthermore, we evaluated a cost-effective approach generating 3’end mRNA sequencing with transcriptome data extracted from seedling without losing prediction ability in comparison to the full-length mRNA sequencing, paving the path for the use of such prediction methods in commercial breeding programs.

**Supplementary Information:**

The online version contains supplementary material available at (10.1186/s12864-022-08337-7).

## Background

Barley (*Hordeum vulgare* L.) is the fourth most important cereal crop in the world (FAOSTAT, http://www.fao.org/faostat/en/) and is used for human nutrition and animal feed [1]. In the context of a growing global population [2], producing sufficient food is a big challenge for agriculture [3]. In addition, climate change is expected to negatively impact global crop production by increasing extreme temperatures and altering rainfall patterns [4]. Thus, high and stable yield in barley is one of the most important breeding goals. However, in addition to directly breeding for yield, the consideration of yield-related characters during the breeding processes proved successful [5]. Leaf angle (LA) e.g. is one of the most important canopy architecture parameters that influence the efficiency of photosynthesis and further affect yield production [6]. In addition, the control of plant height (PH) can be used to reduce yield loss arising from lodging and adaption to variable environments through heading time (HT) alteration impacts yield [7]. Therefore, the use of approaches that help breeders to reliably select for yield and yield-related quantitative traits increases the gain of selection.

Genomic prediction (GP) has emerged as a powerful tool to increase selection gain for complex traits in both livestock and plant breeding programs [8, 9]. This method is based on the idea that the performance of individuals can be predicted from genotypic information using the GP model which was trained on those individuals with both phenotypic and genotypic information. Thus, the genotyped individuals can be preselected before their phenotypes are measured in the field to shorten the breeding cycle as well as to reduce the cost of phenotyping [10].

Typically, single nucleotide polymorphisms (SNP) serve as predictors in GP [11–13]. SNP in gene coding regions can be classified into non-synonymous (nsSNP) and synonymous SNP (sSNP), which differ in their property to change or not the amino acid sequence of a protein. Therefore, these two SNP classes may have different influence on phenotypes. In previous studies, the advantage of using these classes of SNP in comparison to randomly selected SNP for GP was explored in pig [14]. However, they observed that predictive performance of neither nsSNP nor sSNP did significantly differ from those of random SNP for most traits. In addition, Heidaritabar et al. [15] observed that nsSNP did not enhance the performance of GP in chicken. On the other hand, a protein may be able to tolerate an amino acid change due to a nsSNP and still keep its function normal [16]. Therefore, SNP can be grouped using the SIFT algorithm [17] into (1) tolerant SNP (tSNP), which can keep a protein’s function normal; and (2) deleterious SNP (dSNP), which will affect a protein’s function. To the best of our knowledge, the use of tSNP or dSNP as predictor of the phenotypic variation has not yet been compared.

Complex biological processes such as transcription, translation, and biochemical cascades resulting in various metabolites occur between DNA sequence and phenotypes [11], which hamper the predictive power of SNP. In addition, higher-order epistatic effects may contribute to the genetic variance of complex traits [18], which can in most of cases not directly be captured using SNP information [13, 19]. Therefore, prediction ability of phenotypic variation using SNP information for quantitative traits still leaves room for improvement. In the last years, molecular technologies were developed, which allow a cheap and high-throughput gene expression and metabolite profiling [20]. Such data can act as bridge to shorten the biological distance between genotypes and phenotypes and may even capture higher-order epistatic interactions for the prediction of phenotypic variation [21, 22].

Transcription is the first downstream processes after the DNA sequence and, thus, more likely affects the variation of traits compared to SNP. Recently, thanks to technological developments, several studies have proposed to use gene expression (GE) variation as predictor of phenotypic variation in maize [11, 21], rice [22] and barley [23]. While Schrag et al. [21] and Hu et al. [22] used GE assessed from microarray experiments for GP and showed that a considerable proportion of phenotypic variation can be explained by such information, Guo et al. [11] and Weisweiler et al. [23] used mRNA sequencing datasets to predict the performance of phenotypic traits. The advantage of mRNA sequencing compared to microarray experiment is the possibility to extract SNP and small insertions/deletions (INDEL) called sequence variants (SV hereafter), in addition to the quantification of transcript abundance. Furthermore, a single gene can often produce more than one transcript through alternative splicing, which can generate various proteins to regulate the complexity of pathways [24]. These different transcripts of the same gene can be identified using full-length mRNA sequencing. To our knowledge, transcript expression (TE) as predictor in GP has not yet been compared to GE.

Compared to the two previous levels of molecular information (DNA sequence and GE), metabolites (M) have the closest relationship to the expressed phenotype because they are the end-points of upstream biochemical processes [25], and, thus, have a high potential as predictors for GP. Previous studies on the use of metabolites to predict phenotypic traits in *Arabidopsis thaliana*, maize, wheat, and barley reported lower or higher prediction abilities compared to SNP information, depending on the traits and species [11, 21, 26–29]. Gemmer et al. [29] recommended that metabolites cannot be used alone in barley for phenotype prediction. However, the integration of expression and metabolite datasets with SNP information improved prediction abilities in comparison to the benchmark using SNP information in maize [11, 21]. Thus, the integration of several layers of omic datasets such as SV, GE, TE, and M as predictors could outperform benchmark methods and should be evaluated in GP of phenotypic traits in barley.

The objectives of our study were to (i) assess the prediction ability for three yield-related phenotypic traits (LA, PH, and HT) using different omic datasets as single predictors compared to a SNP array, where these omic datasets included different types of sequence variants (SV, dSV, and tSV), different types of transcriptome (expression presence/absence variation-ePAV, GE, and TE) sampled from two tissues, leaf and seedling, and metabolites (M); (ii) investigate the improvement in prediction ability when combining multiple omic datasets information to predict phenotypic variation in barley breeding programs; (iii) explore the predictive performance when using SV, GE, and ePAV from simulated 3’end mRNA sequencing of different lengths as predictors.

## Results

### Heritability

The three phenotypic traits (LA, PH, and HT) were measured for 23 spring barley inbreds in seven environments. The adjusted entry means of the 23 inbreds ranged from 2.52 to 7.07 for LA, 48.75 to 79.75 cm for PH, and 57.31 to 82.23 days for HT (Suppl. Table S1). Heritabilities on an entry mean basis (*H*^2^) were high and similar for LA (0.91) and HT (0.90) and with 0.83 slightly lower for PH. A total of 192 chemical entities were annotated (Suppl. Table S2) and after filtering (see methods), 144 metabolites remained for which the relative abundances were used for further analyses. A total of 101 metabolites were found in databases and, thus, it was possible to assign them according to their chemical features to 12 compound classes, while the remaining 43 metabolites were unknown (Suppl. Table S3). The heritabilities of the metabolites on an entry mean basis ranged from 0 to 0.98 with an average of 0.62 (Suppl. Fig. S1). The classification of the metabolic predictors using different degrees of heritability (0.1 to 0.8 in increments of 0.1) resulted in eight groups with 133, 128, 121, 117, 109, 93, 72 and 45 metabolites, respectively. These groups were then considered for the omic prediction described below.

### Correlation and genetic dissimilarity analyses

**Fig. 1:**
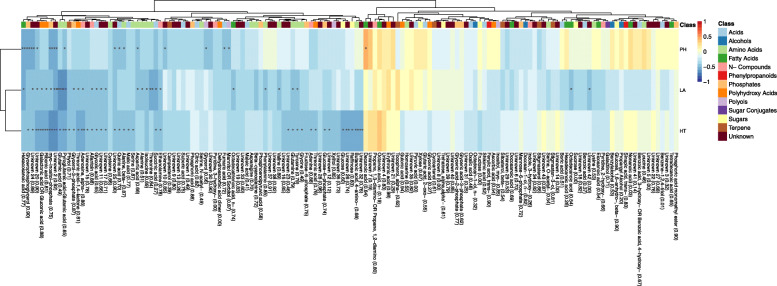
Heatmap of Pearson correlation coefficients calculated between all pairs of the three phenotypic traits and the 144 metabolites. The three phenotypic traits are leaf angle (LA), plant height (PH) and heading time (HT). Correlations marked with ^∗^, ^∗∗^, and ^∗∗∗^ were significant at *P*<0.05, 0.01, and 0.001, respectively. The heritability of each metabolite is given in parentheses after each metabolite’s name

Positive correlations between the three phenotypic traits were observed (Suppl. Fig. S2). Particularly, LA was highly and significantly correlated with HT (0.685 ^∗∗∗^), where the correlation coefficients between PH and HT as well as between PH and LA were with about 0.45 considerably lower. Many metabolites were significantly (*P*<0.05) negatively associated with the assessed phenotypic traits (Fig. 1). For instance, a cluster of some acids, amino acids, and several unknown metabolites was strongly negatively correlated with the three traits. Interestingly, we found that the same metabolites that were significantly correlated with LA were also correlated with HT. This was consistent with the phenotypic correlations between both traits (Fig. 1 and Suppl. Fig. S2).

**Fig. 2:**
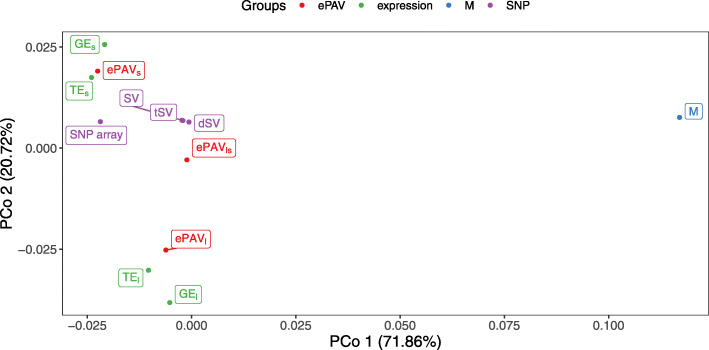
Plot of the first two axes of the principal coordinate analysis for comparison of the similarity between different omic datasets based on generalized procrustes analysis. The omic datasets include SNP array, sequence variants (SV), deleterious sequence variants (dSV), tolerant sequence variants (tSV), gene expression in seedling and leaf (GE _*s*_ and GE _*l*_), transcript expression in seedling and leaf (TE _*s*_ and TE _*l*_), expression presence/absence variation in seedling, leaf and combining both tissues (ePAV _*s*_, ePAV _*l*_, and ePAV _*ls*_), and metabolites (M). The colors show the four groups of omic datasets used in a grid search for integration of multiple predictors (Figure 5). Red represents ePAV, green expression, blue metabolite, and purple SNP and SV predictors

To assess similarity/dissimilarity between these omic datasets, we performed generalized procrustes analysis (GPA) [30] on the resulting principal component analysis (PCA) obtained from each omic dataset. The dissimilarity measurements from GPA were used for principal coordinates analysis (PCoA). The first two PCo accounted for 71.86% and 20.72% of the total variability, respectively (Fig. 2). The first PCo separated the metabolites from the other features while the second PCo tended to differentiate the two tissues, leaf (*l*) and seedling (*s*). GE, TE, and ePAV datasets were similar to each other within the same tissue. This can be explained thereby that the ePAV dataset was derived from GE dataset and the GE dataset was derived from the TE dataset. ePAV _*ls*_ was, as expected, centered between the ePAV from the individual tissues. Although SNP array, SV, dSV, and tSV clustered together, SNP array was more distant from the cluster of dSV, tSV, and SV which almost overlapped. This was due to that dSV and tSV are a subset of SV. This finding indicated that SNP, expression and metabolite features would provide different layers of biological information and might contribute differently and complementarily to the phenotypic variation.

### Omic prediction

**Fig. 3:**
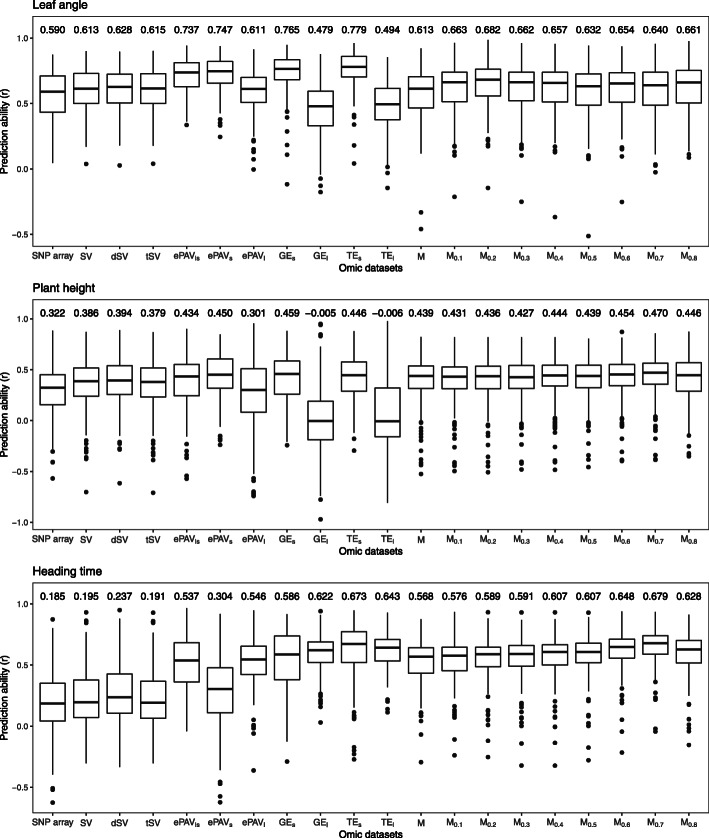
Boxplot of prediction abilities for the three traits, leaf angle, plant height and heading time, based on 22 inbreds using different omic datasets as a single predictor across 200 five-fold cross-validation runs. The values given above each box represent the medians of 200 runs. The omic datasets include SNP array, sequence variants (SV), deleterious sequence variants (dSV), tolerant sequence variants (tSV), gene expression in seedling and leaf (GE _*s*_ and GE _*l*_), transcript expression in seedling and leaf (TE _*s*_ and TE _*l*_), expression presence/absence variation in seedling, leaf and combining both tissues (ePAV _*s*_, ePAV _*l*_, and ePAV _*ls*_), metabolites filtered for their heritability (M, M _0.1_, M _0.2_, M _0.3_, M _0.4_ M _0.5_, M _0.6_, M _0.7_, and M _0.8_)

The prediction ability of the three phenotypic traits using different single predictors was examined through five-fold cross-validation. Regardless of the predictor, the prediction abilities were higher for traits with higher heritabilities (Fig. 3). Prediction abilities based on SV, GE, TE, ePAV, and M datasets were compared to that realized with the SNP array which was used as baseline predictor. The observed median prediction ability based on the SNP array dataset ranged from 0.185 (HT) to 0.590 (LA). The prediction ability of SV extracted from mRNA sequencing dataset was slightly higher than that of SNP array dataset across the three traits. Moreover, the dSV dataset slightly outperformed the SV extracted from mRNA sequencing and the tSV dataset (Fig. 3). Even higher prediction abilities were observed for ePAV, any expression datasets from seedling (GE _*s*_ and TE _*s*_), and metabolite datasets (Fig. 3). The prediction abilities for the ePAV dataset were significantly different among *l*, *s* and *ls*, but not consistently across the three traits (data not shown). ePAV _*ls*_ was chosen as the best compromise across the three traits for further analyses, as it was for none of the three traits in the significance group with the lowest prediction abilities. The TE datasets slightly outperformed the GE datasets for HT and LA, and TE _*s*_ resulted in the highest prediction ability as single predictor for these traits. In contrast, no difference between TE and GE was observed for PH.

To explore whether the heritability of a metabolite affects the prediction performance, eight classes of metabolites based on different degrees of heritabilities served as predictor. The prediction ability increased when the metabolites with lower heritability (<0.1) were not considered (Fig. 3). However, the prediction ability didn’t increase significantly and consistently across the three traits with increasing heritability of the considered metabolites (data not shown). Therefore, we selected the metabolite group for which the highest prediction ability was observed across the three traits (M _0.6_) for further analyses.

**Fig. 4:**
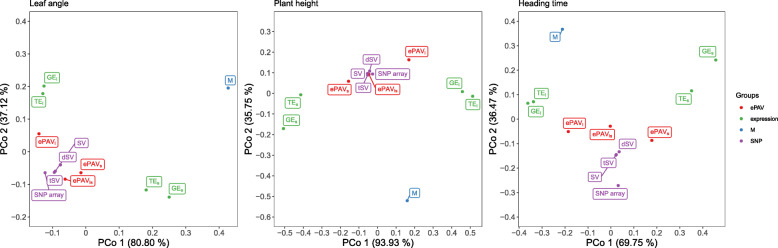
Plot of the first two axes of principal coordinates analysis for comparison of predicted values from different omic datasets as a single predictor based on the median of correlation-based distance across 200 five-fold cross-validation runs for the three traits, leaf angle, plant height and heading time. The omic datasets include SNP array, sequence variants (SV), deleterious sequence variants (dSV), tolerant sequence variants (tSV), gene expression in seedling and leaf (GE _*s*_ and GE _*l*_), transcript expression in seedling and leaf (TE _*s*_ and TE _*l*_), expression presence/absence variation in seedling, leaf and combining both tissues (ePAV _*s*_, ePAV _*l*_, and ePAV _*ls*_), and metabolites (M). The colors show the four groups of omic datasets used in a grid search for integration of multiple predictors (Figure 5). Red represents ePAV, green expression, blue metabolite, and purple SNP and SV predictors

Pearsons correlation coefficients between pairwise predicted values of different omic datasets were calculated, and the correlation-based distance was used for PCoA analysis for each trait. Across the three examined traits, the metabolite feature was clearly separated from the other omics features (Fig. 4), and the predicted values of M were less correlated with those values of the other omic datasets than the other omic datasets among themselves (Suppl. Fig. S3). A similar result was observed between the two tissues, seedling and leaf, which were clearly separated on Fig. 4. In contrast, the predicted values from features that clustered together on Fig. 4, especially SNP array, SV, dSV, tSV, ePAV _*ls*_, were highly correlated (Suppl.Fig. S3).

**Fig. 5:**
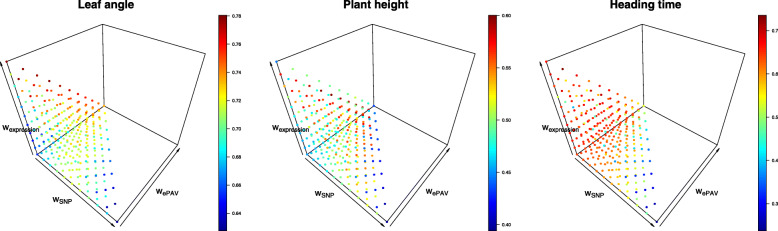
Prediction ability for the three traits, leaf angle, plant height, and heading time, from 22 inbreds for 286 combinations of the joined weighted matrix which differ in the weights of four predictors, deleterious sequence variants (dSV), expression presence/absence variation in combined leaf and seedling (ePAV _*ls*_), transcript expression in seedling (TE _*s*_), and metabolite with a heritability on an entry mean basis >0.6 (M _0.6_). Plotted values represent medians across 200 cross-validation runs

In order to evaluate whether the prediction ability can be improved by combining several predictors, a joined weighted relationship matrix of the single predictors with the highest prediction ability was established and a grid search was used to identify those combinations of dSV, ePAV _*ls*_, TE _*s*_, and M _0.6_ resulting in the highest prediction ability. For the three examined traits, the highest median prediction ability was observed when more than one predictor was used (Fig. 5). Furthermore, the optimal weights of the four predictors to reach the maximal prediction ability differed among the three traits, but the weights of ePAV _*ls*_ and TE _*s*_ were at least 10% and 50%, respectively. However, the optimal weight for M was, except for PH, 0, and the optimal weight for the dSV was 0 for the three traits.

**Fig. 6:**
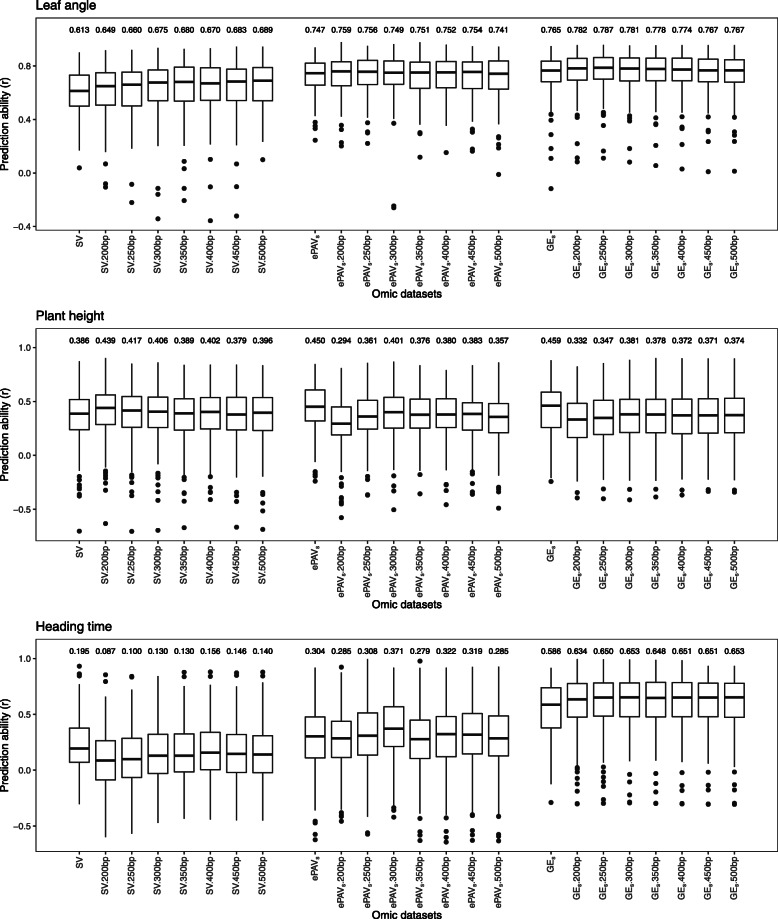
Boxplot of prediction ability for the three traits, leaf angle, plant height and heading time, from 22 inbreds using different omic datasets from simulated 3’end mRNA sequencing with seven length categories (200, 250, 300, 350, 400,450, and 500 bp) as a single predictor across 200 cross-validation runs. The omic datasets from full-length mRNA sequencing are used as a baseline. The values given above each box represent medians of 200 prediction abilities. The omic datasets include sequence variants (SV), gene expression in seedling (GE _*s*_), and expression presence/absence variation in seedling (ePAV _*s*_)

We also assessed the prediction abilities of SV, GE, ePAV from 3’end mRNA sequencing that we simulated from our full-length mRNA sequencing dataset. Depending on the trait, a similar, slightly better or worse median of prediction abilities of SV, GE, ePAV were observed when considering 3’end mRNA sequencing compared to a full-length mRNA sequencing dataset as baseline (Fig. 6). Moreover, we did not observe a systematic trend on the prediction ability when increasing the length of the 3’end mRNA sequencing.

## Discussion

### Ability of different omic features to predict phenotypic traits

Genomic prediction has become a broadly used tool to improve the gain of selection in plant breeding [9]. The current standard procedure of genomic prediction is to use SNP markers generated from SNP array or genotyping by sequencing methods as predictors [12]. However, there are several complicated biological downstream processes such as transcription, translation, and biochemical cascades resulting in various metabolites between DNA sequences and phenotypes [11]. Using predictors that are biologically closer to the phenotypes may increase the prediction ability in genomic predictions. With the development of high-throughput molecular technologies, the availability of such predictors from the genomic, transcriptomic, or metabolomic level is ensured [20]. In this pilot study, we aim to compare different types of omic datasets for their predictive performance in order to prioritize them for their later evaluation in large-scale experiments. We hold that this is true also with only 23 inbreds of our study, especially as these inbreds are representative of and cover most of the genotypic diversity of barley [23].

For the three examined traits, any of the SV information generated from mRNA sequencing (SV, dSV, as well as tSV) resulted in a higher prediction ability compared to the SNP data produced with the 50K SNP array (Fig. 3). This might be explained by the higher number of SV features, as increasing the number of predictors can increase the extent of linkage disequilibrium between SNP and quantitative trait loci (QTL) [23, 31]. In addition, INDEL information was included in the SV, which was not the case in the SNP array. INDEL are one type of genetic variation in living organisms that involve larger DNA fragments than single variants and have been identified in known genes (c.f. [32, 33]). Therefore, they are very usefull for the developpment of functional markers [34] and are expected to cause extreme change in the phenotypes. This could be a further explaination why SV had better predictive performance than SNP array. Our observation is in agreement with the finding that the PCo 1 resulting from the GPA separated clearly SV and SNP array (Fig. 2), which indicates that SV and SNP array provide different information.

SV in gene coding regions can be classified into nsSV and sSV, where the former can change the amino acid sequence of proteins, but not the latter. However, not all amino acid changes lead to significant changes of the protein. This can be explored by the SIFT algorithm in classifying SV into dSV and tSV based on the conversion of amino acid sequences [16], where the former cause a loss of protein function but not the latter. Kono et al. [35] showed that known phenotype-altering variants were more frequently inferred as deleterious than the genome-wide average, and have a higher probability to contribute to phenotypic variation. Thus, we compared the prediction ability of dSV and tSV compared to that of SV across the three traits.

The predicted phenotypic values based on the three different classes of SV were highly correlated with each other (Suppl. Fig. S3), which can be expected because dSV and tSV are a subset of SV and clustered together in the GPA (Fig. 2). However, the prediction ability for the three phenotypic traits using dSV information was slightly higher than using tSV and all SV information, despite the fact that the number of dSV features was far smaller (15,868) than the number of tSV features (117,698) and the total number of SV. This trend of a higher prediction ability for dSV was even more pronounced when adjusting for differences in the number of features by resampling simulations (data not shown). Our finding is in discordance with the results of Do et al. [14] and Heidaritabar et al. [15], who observed no difference between the prediction performance of nsSNP and randomly sampled SNPs. A first explanation for our different findings could be that the former cited studies classified the SNP based on whether they may induce amino acid change or not, whereas our study distinguished tolerant/deleterious SNP. Secondly, the SNP used for GP by Heidaritabar et al. [15] were imputed for all genotypes from a 60K SNP array. This might have hampered the improvement of prediction ability in comparison to our study, which is based on real variant data for all inbreds (except few missing data that were mean-imputed). Our finding indicated that the pre-selection of variants based on their theoreticaly predicted protein function could improve prediction performance of traits, which can be of considerable importance for breeders.

The features derived from the transcriptome datasets (GE, TE, as well as ePAV) led to increased prediction abilities by 62.81% compared to SNP array and even SV on average across the three traits and two tissues. This finding was inconsistent with the results of previous studies [11, 21], who observed that the prediction abilities based on transcriptomic datasets were a little lower (5.30% and 0.03%) than those based on genomic information averaged across the examined traits. This difference might be caused by the complex genetic architectures of traits evaluated and tissue sampled in the studies cited above. However, the use of transcriptomic datasets as predictors still had reasonable prediction abilities in the former studies, which is in accordance with our results and can be explained by the fact that with such datasets expression levels can be quantified and physiological epistasis even captured.

A single gene can encode multiple distinct transcripts through alternative splicing, which allows organisms to increase the protein diversity based on the same set of genes [36], and therefore could lead to more phenotypic variation. As a consequence, a higher prediction ability could be expected for phenotypic traits predicted from TE compared to GE information. This was confirmed by our findings (Fig. 3), and suggests that TE information might be more efficient than GE information in predicting the performance of traits when the full-length mRNA sequencing has been performed.

All the datasets generated by mRNA sequencing from seedling were well separated from those from leaf (Fig. 2). Similarly, the correlation between predicted patterns based on the transcriptomic dataset of the two tissues was low (Fig. 4 and Suppl. Fig. S3), which indicated that different types of tissue offer dissimilar information concerning the phenotypic variation and influence the prediction ability. In general, the prediction ability was considerably higher for the datasets from seedling in comparison with the datasets from leaf on average across the three traits (Fig. 3). This might be explained by the fact that more diverse genes are expressed in seedling than in leaf.

Only for HT, expression information from leaf (GE _*l*_, TE _*l*_) achieved the same level of prediction ability as that from seedling. One explanation for this finding might be that HT is triggered by environmental factors in later developmental stages and therefore the causal expression features for this trait are more likely to be revealed in leaf than in early developmental stages like seedling.

A total of 53 of the 144 metabolites quantified in our study were significantly correlated with at least one of the three phenotypic traits (Fig. 1). This suggests that the metabolites can be used for selection for phenotypes. In addition, the metabolite feature was clearly separated from the other features in the similarity/dissimilarity analysis (Fig. 2). More importantly, the correlations between the predicted values based on metabolic feature and other omic datasets were low, and lower than the correlation between different other omic datasets (Suppl. Fig. S3). This finding suggested that the metabolites can provide another biological layer of information to capture the phenotypic variation. We observed across the three traits that prediction abilities based on metabolites were considerably higher compared to SNP or SV information (Fig. 3). This finding is in contradiction to results of previous studies [11, 29] who revealed considerably lower prediction ability using metabolites as predictor. This might be caused by the high accuracy of the metabolite assessment used in our study. The average heritability on an entry mean basis across 144 metabolites was with about 0.62 considerably higher than that observed by Guo et al. [11] with 0.49 and Gemmer et al. [29] with 0.26. This aspect was studied further by leaving out those metabolites with heritabilities <0.1. This resulted in an increased prediction ability for all traits, which suggested that higher accuracy of metabolites can bring stable information in the prediction of phenotypes.

Generally, (di)-similarity between (1) different omic datasets (Fig. 2) and also between (2) the correlation between predicted phenotypic traits based on different omic datasets (Fig. 4 and Suppl. Fig. S3) was observed in our study. This suggested complementation between different biological perspectives to the phenotypic variation. Therefore, combining predictors covering different layers of biological information in an integrative model could have an advantage over the GP model based on single predictors, and was examined in our study.

### Increasing prediction abilities by combining multiple predictors

In this study, a grid search was used to identify those combinations of dSV, ePAV _*ls*_, TE _*s*_, and M _0.6_ in the joined weighted relationship matrix of GBLUP model maximizing the prediction ability. The highest prediction ability across the three examined traits was observed when more than one predictor was used and, for each of the three traits, without the contribution of the dSV (Fig. 5). This finding might be explained by the fact that transcriptome and metabolome information are closer to phenotypes than gene information according to the central dogma of molecular biology, and can capture together more genetic variation and physiological epistasis caused by complicated networks and interactions between genes than when using only one single predictor [11].

On the other hand, even if a higher prediction ability for all three examined traits was observed if more than one predictor was used (Fig. 5), the optimal weight of each component in the joined weighted relationship matrix depended highly on the traits. For instance, metabolite information was needed to obtain the highest prediction ability for PH, but not for the other traits. Transcriptome was the most important component, but the weight ranged from 0.5 to 0.9 across the three traits. From the physiological point of view, this might be explained by the different genetic architectures of the different traits and their exposure to different environments at different developmental stages and tissues. We observed the tendency that for traits with a lower heritability more different omic predictors were needed to result in the highest prediction ability. Further research on traits with high genetic complexity and low heritability such as yield is needed to test this hypothesis.

### Summary: application in breeding programs

The results of our study suggested that combining the information of SV, expression, as well as metabolite dataset into genomic prediction models can improve the prediction ability of phenotypic traits. Especially, the expression datasets were the most important components for this improvement (Fig. 5). To be implemented in breeding programs, such datasets have to be created approximately at the costs of one traditional phenotyping unit (c.f. [37]). This implies that the datasets of SV, gene expression, and metabolite are sampled from one tissue, to avoid the cost of multiple sampling at several stages. The goal of this study was to compare predictors for their ability to predict phenotypic traits. The results of our study indicate that the higher and more stable predictive performance across traits can be achieved from gene and transcript expression gained on seedling samples. Seedling samples combine both aptitude in reaching a high prediction ability but can be also generated in a cost-effective and high-throughput manner. Thus, they are recommended as the best tissue to predict the variation of phenotypes in barley populations. However, for other crops such as tuber crops, different approaches and tissues might be needed, which requires further research.

The limited budget available in practical breeding programs for full-length mRNA sequencing hampers the use of such approaches. Instead, 3’end mRNA sequencing could be a cost-effective alternative method to obtain transcriptome information. For 3’end mRNA sequencing, only 50-800bp at the 3’end of the genes are sequenced. Interestingly, we observed that the prediction abilities of SV, GE, ePAV from simulated 3’end mRNA sequencing were on average across the three traits similar to those from the full-length mRNA sequencing (Fig. 6). Therefore, our finding suggested that transcriptome data can be generated from the 3’end mRNA sequencing without losing prediction ability in comparison to the full-length mRNA sequencing, paving the path for the use of such prediction methods in commercial breeding programs.

Although this study is based on a limited number of barley inbreds, it can be considered as a pilot research showing how different omic datasets can improve prediction of phenotypic variation and will open the path to perform such analysis on a bigger scale, e.g. on segregating populations derived from the 23 inbreds [38].

## Materials and methods

### Plant materials and phenotypic data collection

This study was based on 23 spring barley inbreds which were selected from a worldwide collection [39] to maximize phenotypic and genotypic diversity [23]. The 23 inbreds were planted as replicated checks in a field experiment laid out as an augmented row-column design. The experiment was performed in seven agro-ecologically diverse environments (Cologne from 2017 to 2019, Mechernich and Quedlinburg from 2018 to 2019) in Germany in which the checks were replicated 10 to 21 times per environment. At each environment, three yield-related phenotypic traits were assessed. The leaf angle (LA) was scored on a scale from 1 (erect) to 9 (very flat) on four-week-old plants. The heading time (HT) was recorded as days after planting. Furthermore, the plant height (PH, cm) was measured after heading (only assessed in Cologne and Mechernich).

### Omic datasets

#### Metabolite profiling

The metabolite profiling of our study was based on leaf samples collected for the 23 barley inbreds with quadruplicates in a greenhouse experiment, where no phenotypic traits were assessed. Seeds of the 23 spring barley inbreds were sown in controlled conditions with 16 hours light and eight hours dark at 22 ^∘^C. Plantlets were cultivated for two weeks and then moved to vernalisation in a growth chamber. After five weeks of vernalisation, the plants were repotted and returned to the greenhouse. After one week, one 3 x 1cm piece of the central part of the youngest fully developed leaf was harvested from two plants of the same inbred, pooled, and immediately flash frozen in liquid nitrogen. The collection of all samples was done within one hour to minimize the variation due to circadian rhythms. Each of the 92 samples was analyzed one time via gas chromatography-mass spectrometry (GC-MS) using an adapted protocol from Lisec et al. [40]. Metabolites were extracted from 45-55 mg frozen mortared samples with 1.5 ml of a 1:2.5:1 H_2_O:methanol:chloroform (v:v:v) mixture pre-cooled to −20 ^∘^C, then mixed on a rotator for 10 min and centrifuged at 20,000 g for 2 min (both at 4 ^∘^C). A total of 30 *μ*l of the supernatant were dried completely in a vacuum concentrator and derivatized in two steps via an MPS-Dual-head autosampler (Gerstel): (1) with 10 *μ*l methoxyamine hydrochloride (Acros organics; freshly prepared at 20 mg/ml in pure pyridine (Sigma-Aldrich)) and shaking at 37 ^∘^C for 90 min, (2) adding 90 *μ*l N-Methyl-N-(trimethylsilyl)trifluoroacetamide (MSTFA; Macherey-Nagel) and shaking at 37 ^∘^C for 30 min. After incubation for 2 hours at room temperature, 1 *μ*l of derivatized compounds was injected at a flow of 1 ml/min with an automatic liner exchange system in conjunction with a cold injection system (Gerstel) in splitless mode (ramping from 50 ^∘^C to 250 ^∘^C at 12 ^∘^C/s) into the GC. Chromatography was performed using a 7890B GC system (Agilent Technologies) with a 30 m long, 0.25 mm internal diameter, HP-5MS column with 5% phenyl methyl siloxane film (Agilent 19091S-433). The oven temperature was held constant at 70 ^∘^C for 2 min and then ramped at 12.5 ^∘^C/min to 320 ^∘^C at which it was held constant for 5 min; resulting in a total run time of 27 minutes.

Metabolites were ionized with an electron impact source at 70V and 200 ^∘^C source temperature and recorded in a mass range of m/z 60 to m/z 800 at 20 scans per second with a 7200 GC-QTOF (Agilent Technologies). Raw data files exported from MassHunter Qualitative (v b07, Agilent Technologies) in the mzData format (*mzdata.xml) were converted to the NetCDF format (*.cdf) and baseline-corrected via MetAlign (v 041012, [41]) using default parameters. Baseline-correction was visually inspected using OpenChrom (v 1.3.0, [42]). Quantitative analysis of GC-MS-based metabolite profiling experiments was then performed using TagFinder (v 4.1, [43]). After evaluating the uniqueness and linearity of each fragment, the aggregated fragment intensity was calculated as the average of the maximum scaled fragment intensity. For relative quantification, aggregated fragment intensities of the compounds were normalized to those of the internal standard ribitol (Sigma-Aldrich) which was added to the extraction buffer. Mass spectral annotation was manually supervised using the Golm Metabolome Database mass-spectral library (http://gmd.mpimp-golm.mpg.de/download/) after conversion of absolute time in retention indices [44]. The raw data, details of the quantification and annotation steps, and the processed metabolite profiles are available (https://www.ebi.ac.uk/metabolights/MTBLS1561). The compounds corresponding to contaminations, siloxane, ribitol, and dimethylphenylalanine were removed. Furthermore, if several compounds were identified as the same metabolite, the one with the greatest heritabilty, for which the calculation is described below, was retained.

#### SNP genotyping, RNA extraction, sequencing, and quantification of gene expression

The Illumina 50K barley SNP array [45] was used to genotype the 23 inbreds of our study [23]. This dataset is designated in the following as SNP array.

mRNA was extracted from leaf and seedling samples of the 23 inbreds as described earlier by Weisweiler et al. [23]. 46 polyA enriched RNA libraries were prepared at the Max Planck Genome Centre Cologne (https://mpgc.mpipz.mpg.de/home/). In addition, two tissue samples of one of the inbreds and one tissue sample of two other inbreds had to be removed during the data cleaning process. Reads were trimmed, adapter and low quality regions were removed. Afterwards, reads were mapped using HISAT2 (version 2.0.5) [46] to the Morex referene sequence version 1 [47]. Transcript calling was performed with StringTie (version 2.1.3) [48]. Newly identified and annotated genes were included to the dataset as described by Weisweiler et al. [23]. The expression data for the 23 inbreds was separated into gene expression and transcript expression data. The expression quantified as fragments per kilobase of exon model per million fragments mapped (FPKM) was measured for every transcript of a gene, resulting in one FPKM-value per gene and the corresponding FPKM-value for each transcript of a gene. The FPKM-values of genes and transcripts are designated in the following as GE and TE, where the indexes *l* and *s* were used to separate the leaf (GE _*l*_, TE _*l*_) and seedling (GE _*s*_, TE _*s*_) samples. For further details see Weisweiler et al. [23].

#### Determination of ePAV

For each tissue separately, a presence call was made for each inbred-gene combination in the matrix of presence/absence calls, if GE >0 and an absence call if GE = 0. No presence/absence call (“NA”) was made for the inbreds with 0 <*GE*<10% of the maximum value of GE for a gene-tissue combination (cf. [49]). Tissue specific ePAV calls were combined to an across tissue ePAV call as described in detail by Weisweiler et al. [23]. The ePAV detection procedure resulted in three ePAV data sets, namely ePAV leaf (ePAV _*l*_), ePAV seedling (ePAV _*s*_), and one across both tissues (ePAV _*ls*_).

#### Sequence variant calling

Variant calling of SNP and small INDEL and their filtering was performed with samtools (version 1.11) and bcftools (version 1.10.2) as described by Weisweiler et al. [23], and the dataset is designed in the following as SV. SIFT4G (version 2.4) was used to annotate and predict tolerant and deleterious variants. The prediction was done based on the conversion of amino acid sequences [16]. Amino acid substitutions were classified according to their effect on the protein functions and were predicted as tolerant if the score was >0.05 and as deleterious if the score was <= 0.05. The SIFT4G database was build based on the uniref 90 database (downloaded 2020/04/29) and the Morex reference sequence version 1 [47] with the tool SIFT4_Create_Genomic_DB.

#### Simulation of 3’end mRNA sequencing

For the simulation of 3’end mRNA sequencing, GE _*s*_ was only measured based on the last 200, 250, 300, 350, 400, 450, and 500 bp at the 3’end of each gene. To the same reduced set of sequence data, the ePAV detection procedure and the SV calling procedure has been applied resulting in seven different GE, ePAV, and SV datasets.

### Statistical analyses

#### Adjusted entry means, variance components, and heritability

Based on visual inspections of quantile-quantile (Q-Q) plots of residuals as well as residuals vs. fitted values plots, phenotypic outliers were removed. Each of the phenotypic traits was then analysed across the environments using the following mixed model:

1 $$\begin{array}{*{20}l} y_{ijk} = \mu + E_{j} + G_{i} + (G \times E)_{ij} + \varepsilon_{ijk},  \end{array} $$

where *y*_*ijk*_ was the observed phenotypic value for the *i*^*th*^ genotype at the *j*^*th*^ environment within the *k*^*th*^ replication, *μ* the general mean, *G*_*i*_ the effect of the *i*^*th*^ inbred, *E*_*j*_ the effect of the *j*^*th*^ environment, (*G*×*E*)_*ij*_ the interaction between the *i*^*th*^ inbred and the *j*^*th*^ environment, and *ε*_*ijk*_ the random error. To estimate adjusted entry means for all inbreds, *G*_*i*_ was treated as fixed and the other effects as random. As the samples for metabolites were collected from one environment, the model [1] was reduced to:

2 $$\begin{array}{*{20}l} y_{ik} = \mu + G_{i} + \varepsilon_{ik}, \end{array} $$

where *y*_*ik*_ was the observed metabolite for the *i*^*th*^ inbred within the *k*^*th*^ replication, and *ε*_*ik*_ the random error. The resulting adjusted entry means of phenotypic traits and metabolites for each inbred were used in further analyses, where the adjusted entry means of metabolites were designated as M.

To estimate the genetic variance ($\sigma ^{2}_{G}$), model (1) and (2) were used but considering *G*_*i*_ as random. The heritability on an entry mean basis for the phenotypic traits and metabolites was then calculated as $ H^{2} = \sigma _{G}^{2}/(\sigma _{G}^{2} + \bar {\nu }/2)$, where $\bar {\nu }$ was the mean variance of difference between two adjusted entry means [50].

#### Prediction of phenotypic traits from multi-omic datasets

The performance to predict phenotypic variation of different types of predictors: (1) SNP array, (2) sequence variants (SV), (3) deleterious sequence variants (dSV), (4) tolerant sequence variants (tSV), (5) ePAV _*s*_, (6) ePAV _*l*_, (7) ePAV _*ls*_, (8) gene expression in seedling (GE _*s*_), (9) gene expression in leaf (GE _*l*_), (10) transcript expression in seedling (TE _*s*_), (11) transcript expression in leaf (TE _*l*_), (12) metabolite (M), was compared based on the most stable and widely used model in GP, genomic best linear unbiased prediction (GBLUP) model [51], which can be described as

3 $$\begin{array}{*{20}l} \mathbf{y} = \mathbf{1} \mu + \mathbf{Zu} + \boldsymbol{\varepsilon} \end{array} $$

where **y** is the vector of the adjusted entry means of the examined trait, **1** the unit vector, *μ* the general mean, **Z** the incidence matrix of genotypic effects, and **u** the vector of genotypic effects that are assumed be normal distributed with $N(0, \mathbf {G}\sigma ^{2}_{u})$, in which **G** denotes the relationship matrix between inbreds and $\sigma ^{2}_{u}$ the genetic variance. In addition, ***ε*** is the vector of residuals following a normal distribution $N(0, \mathbf {I} \sigma ^{2}_{e})$. In this study, only additive effects were modeled.

For each of the above mentioned omic dataset, the monomorphic features and the features with missing rates >0.2 have been filtered out. **W** was defined as a matrix of feature measurements for the respective omic dataset that is designated in the following as predictor. The dimensions of **W** were the number of barley inbreds (*n*) times the number of features in the corresponding predictor (m) (Table 1). Because of genotyping problems for one of the inbreds, 22 inbred lines were used for further analyses (*n*=22).

**Table 1:** The number of features and the abbreviations for each omic dataset used in this study

Omic dataset	Abbreviation	Number of features
50K SNP array	SNP array	38,285
Sequence variants	SV	133,566
Deleterious sequence variants	dSV	15,868
Tolerant sequence variants	tSV	117,698
Expression presence/absence variation in seedling	ePAV _*s*_	27,445
Expression presence/absence variation in leaf	ePAV _*l*_	26,653
Expression presence/absence variation in combining leaf and seedling	ePAV _*ls*_	36,235
Gene expression in seedling	GE _*s*_	67,844
Gene expression in leaf	GE _*l*_	60,888
Transcript expression in seedling	TE _*s*_	250,490
Transcript expression in leaf	TE _*l*_	220,749
Metabolites	M	144

For each predictor, the additive relationship matrix **G** was defined as $\mathbf {G} = \frac {\mathbf {W^{*}W^{*}}^{T}}{m}$, where *W*^∗^ is a matrix of feature measurement for the respective predictor, whose columns are centered and standardized to unit variance of **W**, and $\mathbf {W^{*^{T}}}$ is the transpose of *W*^∗^. In addition, to assess the impact of the heritability of a metabolite on the prediction performance, only those metabolites with a heritability on an entry mean basis higher than *t*, where *t* varied from 0.1 to 0.8 in increments of 0.1, were considered, and the datasets were designated as M _0.1_, M _0.2_, M _0.3_, M _0.4_ M _0.5_, M _0.6_, M _0.7_ and M _0.8_.

In order to understand whether the different omic datasets can capture similar genetic information, Pearsons correlation coefficients between pairwise predicted values of different omic datasets were calculated. Subsequently, 1 − the correlation coefficients among all pairs of predictors was used as the correlation-based distance in a PCoA. Furthermore, to investigate the performance of a joined weighted relationship matrix [21] to predict phenotypic variation, the matrices **G** in model (3) of four predictors were weighted and summed up to one joined weighted relationship matrix, where we varied:

1. the weight of SNP (*w*_*SNP*_): the weight of the most representative SNP datasets was determined as the one from the SNP array, SV, tSV, or dSV which has the most stable prediction performance across the three traits (dSV).
2. the weight of ePAV (*w*_*ePAV*_): the weight of the most representative ePAV datasets was determined as the one from ePAV _*ls*_, ePAV _*s*_, or ePAV _*l*_ which has most stable prediction performance across the three traits (ePAV _*ls*_).
3. the weight of expression (*w*_*expression*_): the weight of the most representative of the expression datasets was determined as the one from GE _*s*_, GE _*l*_, TE _*s*_, or TE _*l*_ which has most stable prediction performance across the three traits (TE _*s*_).
4. the weight of metabolite (*w*_*M*_,1−*w*_*SNP*_−*w*_*ePAV*_−*w*_*expression*_): the weight of the most representative metabolite datasets was determined as the one from M, M _0.1_, M _0.2_, M _0.3_, M _0.4_ M _0.5_, M _0.6_, M _0.7_, or M _0.8_ which has most stable prediction performance across the three traits (M _0.6_).

A grid search, varying any weight (*w*) from 0 to 1 in increments of 0.1, resulted in 286 different combinations of joined weighted relationship matrix, where the summation of four weights in each combination must be equal to 1. In addition, the performance of SV, GE _*s*_, and ePAV _*s*_ from simulated 3’end mRNA sequencing of different length as described above was explored.

Five-fold cross-validation was used to assess the model performance. Prediction abilities were obtained by calculating Pearson correlations between observed (*y*) and predicted $(\hat {y})$ adjusted entry means in the validation set of each fold. The median prediction ability across the five folds within each replicate was calculated and the median of the median across the 200 replicates was used for further analyses.

#### Correlation and genetic similarity analyses

Correlations among the three phenotypic traits, and between the three phenotypic traits and the individual metabolites were measured as Pearson correlation coefficient. Principal component analysis (PCA) was performed on each omic dataset (SNP array, SV, dSV, tSV, ePAV _*s*_, ePAV _*l*_, ePAV _*ls*_, GE _*l*_, GE _*s*_, TE _*s*_, TE _*l*_, and M). To evaluate similarity/dissimilarity among the various datasets, generalized procrustes analysis (GPA) [30] was performed based on the PCA results. Subsequently, 1 − the procrustes similarity indexes among all pairs of omic datasets was used as dissimilarity measurements in a principal coordinates analysis (PCoA).

All analyses have been performed using the statistical software R [52].

## Supplementary Information

**Additional file 1** Supplemental Materials.

**Additional file 2** Supplementary Table S1.

**Additional file 3** Supplementary Table S2.

## Data Availability

The sequencing datasets have been deposited in the NCBI Sequence Read Archive (SRA) under accession PRJNA534414. The metabolite dataset have been deposited in the MetaboLights (https://www.ebi.ac.uk/metabolights/MTBLS1561). The phenotypic dataset of the adjusted entry means for the three traits can be found in Supplementary Table S1. The annotation and abundance of metabolites can be found in Supplementary Table S2.

## Abbreviations

GP: Genomic prediction
GBLUP: Genomic best linear unbiased prediction
SNP: Single nucleotide polymorphisms
INDEL: Insertions/deletions
nsSNP: Non-synonymous single nucleotide polymorphisms
sSNP: Synonymous single nucleotide polymorphisms
tSNP: Tolerant single nucleotide polymorphisms
dSNP: Deleterious single nucleotide polymorphisms
SV: Sequence variants
dSV: Deleterious sequence variants
tSV: Tolerant sequence variants
*l*: Leaf
*s*: Seedling
ePAV: Expression presence/absence variation
ePAV _*s*_: Expression presence/absence variation in seedling
ePAV _*l*_: Expression presence/absence variation in leaf
ePAV _*ls*_: Expression presence/absence variation in combining leaf and seedling
GE: Gene expression
GE _*s*_: Gene expression in seedling
GE _*l*_: Gene expression in leaf
TE: Transcript expression
TE _*s*_: Transcript expression in seedling
TE _*l*_: Transcript expression in leaf
M: Metabolites
QTL: Quantitative trait loci
GPA: Generalized procrustes analysis
PCA: Principal component analysis
PCoA: Principal coordinates analysis
LA: Leaf angle
PH: Plant height
HT: Heading time
GC-MS: Gas chromatography-mass spectrometry
FPKM: Fragments per kilobase of exon model per million fragments mapped
w _*SNP*_: Weight of single nucleotide polymorphisms
w _*ePAV*_: Weight of expression presence/absence variation
w _*expression*_: Weight of expression
w _*M*_: Weight of metabolite
