# Supplementary material for: Improvement of prediction ability by integrating multi-omic datasets in barley
Source: BMC Genomics. 2022 Mar 12;23:200. doi: 10.1186/s12864-022-08337-7 (PMC8917753; doi:10.1186/s12864-022-08337-7)
Supplement: Supplementary file 1 — Additional file 1 Supplemental Materials. [file 12864_2022_8337_MOESM1_ESM.pdf]

# Improvement of Prediction Ability by Integrating Multi-omic Datasets in Barley

Po-Ya Wu[1], Benjamin Stich[1,4], Marius Weisweiler[1], Asis Shrestha[1], Alexander Erban[2], Philipp Westhoff[3,4], and Delphine Van Inghelandt[1,*]

[1]Institute of Quantitative Genetics and Genomics of Plants, Heinrich Heine University, 40225 Düsseldorf, Germany
[2]Department of Molecular Physiology, Max-Planck-Institute of Molecular Plant Physiology, 14476 Potsdam-Golm, Germany
[3]Institute of Plant Biochemistry, Heinrich Heine University, 40225 Düsseldorf, Germany
[4]Cluster of Excellence on Plant Sciences (CEPLAS), Heinrich Heine University, 40225 Düsseldorf, Germany
[*]Corresponding author: Delphine Van Inghelandt, inghelan@hhu.de

December 8, 2021

[*]Corresponding author: Delphine Van Inghelandt: inghelan@hhu.de, Tel: **49-211/81-13396

# SUPPLEMENTAL MATERIALS

## List of Supplemental Tables

- Table S1: The adjusted entry means of the 23 inbreds for the three traits, leaf angle (LA), plant height (PH) and heading time (HT).

- Table S2: The information of 192 chemical entries and their relative abundance for each inbred.

- Table S3: The classification of the 144 metabolites based on their chemical properties.

## List of Supplemental Figures

- Figure S1: Distribution of heritabilities ($H^2$) for the 144 metabolites. The average (0.62) is indicated as red vertical line.

- Figure S2: Pearson correlation coefficients calculated between all pairs of adjusted entry means of the three phenotypic traits.

- Figure S3: Heatmap of correlation coefficients calculated between all pairs of the predicted values of omic datasets for the three traits, leaf angle, plant height and heading time, across 200 five-fold cross-validation runs. The values given in each cell represent the medians of 200 runs. The omic datasets include SNP array, sequence variants (SV), deleterious sequence variants (dSV), tolerant sequence variants (tSV), gene expression in seedling and leaf ($GE_l$

and $GE_s$), transcript expression in seedling and leaf ($TE_l$ and $TE_s$), expression presence/absence variation in seedling, leaf and combining both tissues ($ePAV_s$, $ePAV_l$, and $ePAV_{ls}$), and metabolites (M)

Table S1: The adjusted entry means of the 23 inbreds for the three traits, leaf angle (LA), plant height (PH) and heading time (HT).

`Suppl_Table_S1_AEM_3traits_23_inbreds.csv`

Table S2: The information of 192 chemical entries and their relative abundance for each inbred.

`Suppl_Table_S2_192_analytes_information.csv`

Table S3: The classification of the 144 metabolites based on their chemical properties.

| Metabolites | Number |
|---|---|
| Amino Acids | 22 |
| Acids | 16 |
| Phosphates | 11 |
| Fatty Acids | 10 |
| Polyhydroxy Acids | 9 |
| N-Compounds | 8 |
| Alcohols | 4 |
| Sugars | 7 |
| Terpene | 7 |
| Polyols | 4 |
| Phenylpropanoids | 2 |
| Sugar Conjugates | 1 |
| Unknown | 43 |

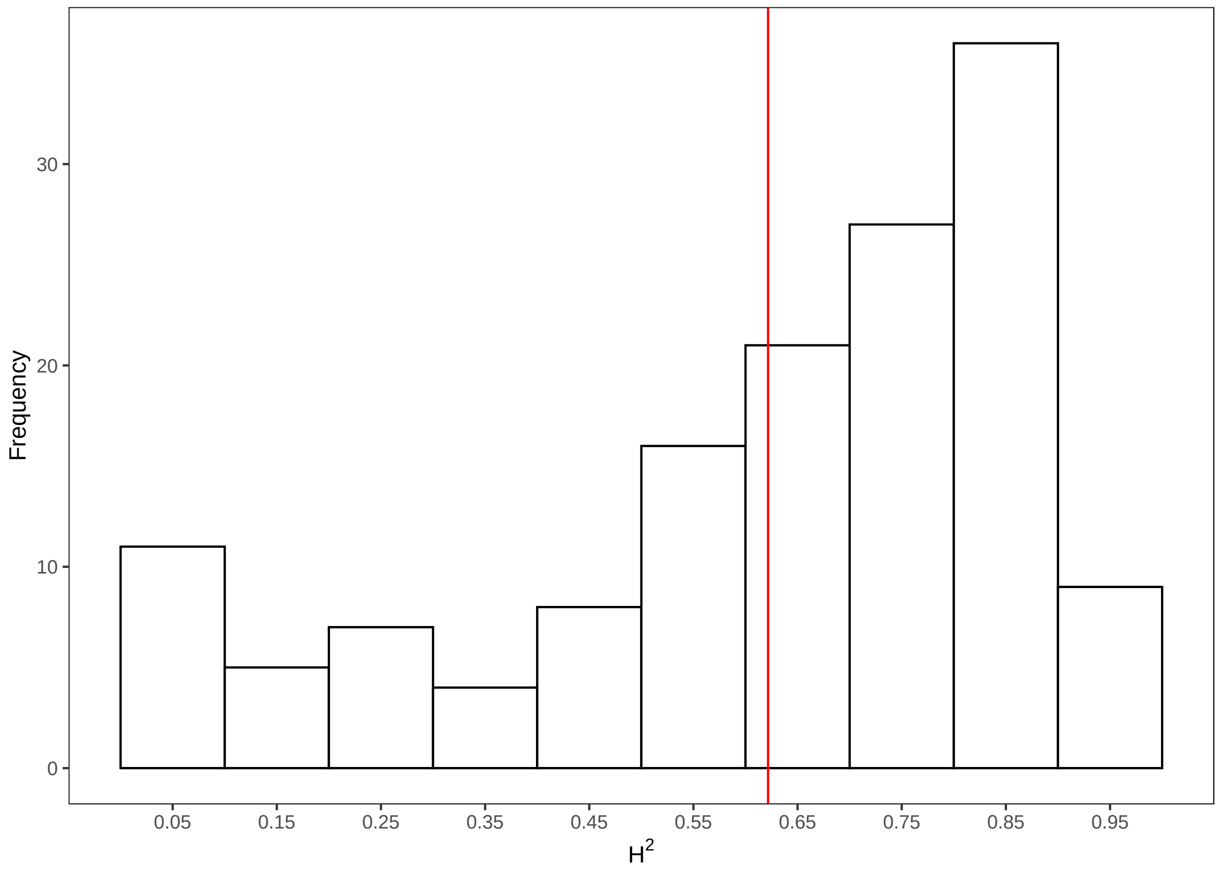


Figure S1: Distribution of heritabilities ($H^2$) for the 144 metabolites. The average (0.62) is indicated as red vertical line.

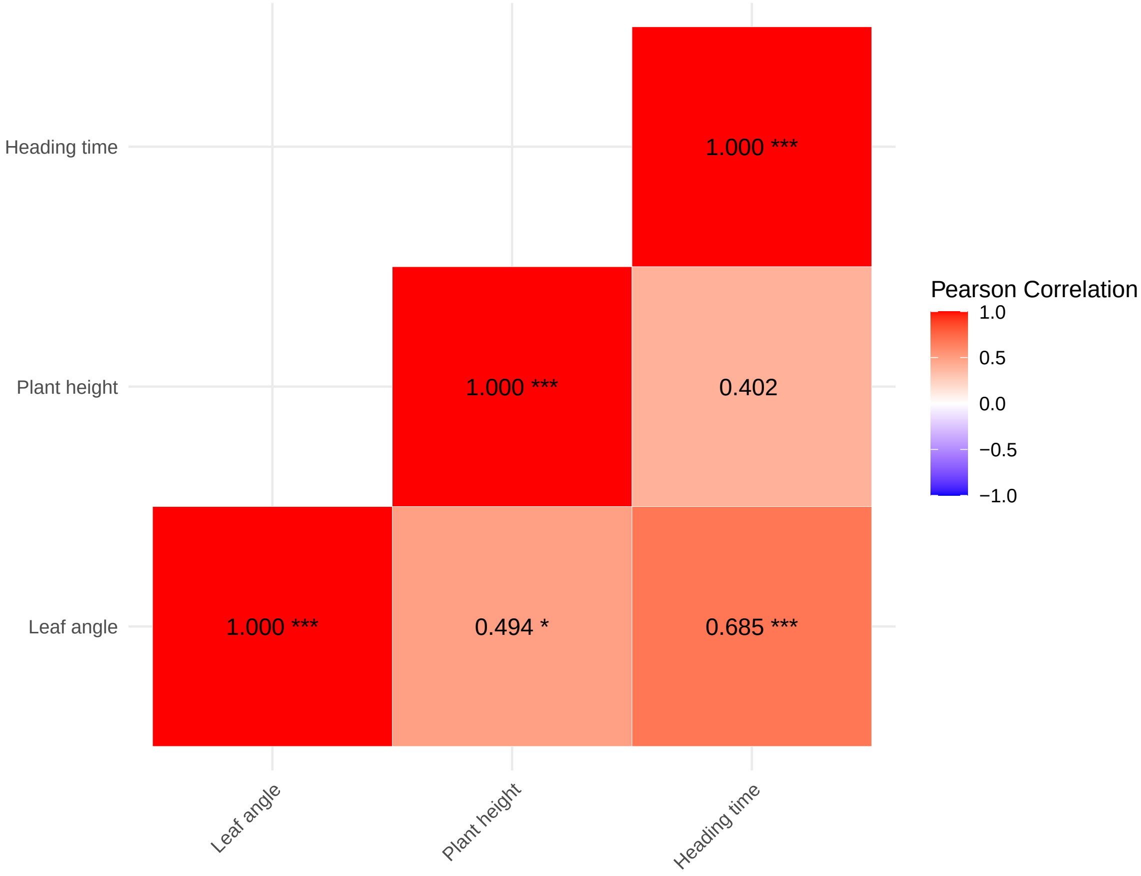


Figure S2: Pearson correlation coefficients calculated between all pairs of adjusted entry means of the three phenotypic traits.

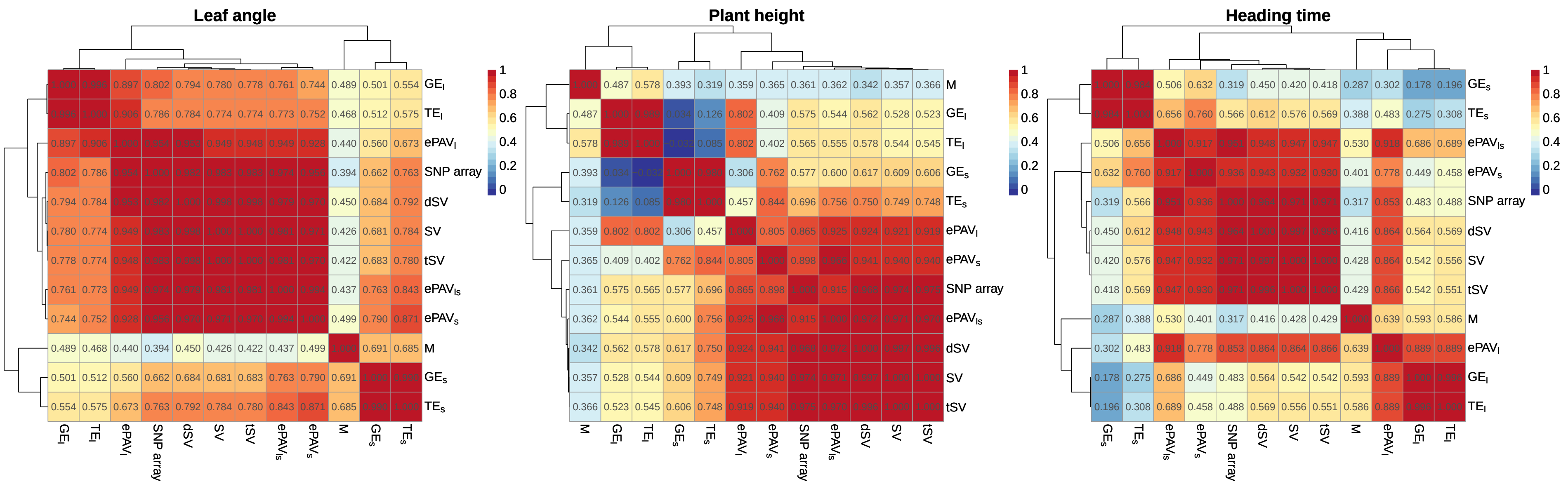

Figure S3: Heatmap of correlation coefficients calculated between all pairs of the predicted values of omic datasets for the three traits, leaf angle, plant height and heading time, across 200 five-fold cross-validation runs. The values given in each cell represent the medians of 200 runs. The omic datasets include SNP array, sequence variants (SV), deleterious sequence variants (dSV), tolerant sequence variants (tSV), gene expression in seedling and leaf ($GE_l$ and $GE_s$), transcript expression in seedling and leaf ($TE_l$ and $TE_s$), expression presence/absence variation in seedling, leaf and combining both tissues ($ePAV_s$, $ePAV_l$, and $ePAV_{ls}$), and metabolites (M).
